# Effects of Apolipoprotein E Genotype on the Off-Line Memory Consolidation

**DOI:** 10.1371/journal.pone.0051617

**Published:** 2012-12-12

**Authors:** De-Yi Wang, Xiu-Jie Han, Su-Fang Li, Dong-Qiang Liu, Chao-Gan Yan, Xi-Nian Zuo, Chao-Zhe Zhu, Yong He, Vesa Kiviniemi, Yu-Feng Zang

**Affiliations:** 1 State Key Laboratory of Cognitive Neuroscience and Learning, Beijing Normal University, Beijing, China; 2 Department of Neurology, Anshan Changda Hospital, Anshan, Liaoning, China; 3 Center for Cognition and Brain Disorders and The Affiliated Hospital, Hangzhou Normal University, Hangzhou, Zhejiang, China; 4 The Nathan Kline Institute for Psychiatric Research, Orangeburg, New York, United States of America; 5 Laboratory for Functional Connectome and Development, Key Laboratory of Behavioural Science, Institute of Psychology, Chinese Academy of Sciences, Beijing, China; 6 Department of Diagnostic Radiology, University Hospital of Oulu, Oulu, Finland; “Mario Negri” Institute for Pharmacological Research, Italy

## Abstract

Spontaneous brain activity or off-line activity after memory encoding is associated with memory consolidation. A few recent resting-state functional magnetic resonance imaging (RS-fMRI) studies indicate that the RS-fMRI could map off-line memory consolidation effects. However, the gene effects on memory consolidation process remain largely unknown. Here we collected two RS-fMRI sessions, one before and another after an episodic memory encoding task, from two groups of healthy young adults, one with apolipoprotein E (APOE) ε2/ε3 and the other with APOE ε3/ε4. The ratio of regional homogeneity (ReHo), a measure of local synchronization of spontaneous RS-fMRI signal, of the two sessions was used as an index of memory-consolidation. APOE ε3/ε4 group showed greater ReHo ratio within the medial temporal lobe (MTL). The ReHo ratio in MTL was significantly correlated with the recognition memory performance in the APOE ε3/ε4 group but not in ε2/ε3 group. Additionally, APOE ε3/ε4 group showed lower ReHo ratio in the occipital and parietal picture-encoding areas. Our results indicate that APOE ε3/ε4 group may have a different off-line memory consolidation process compared to ε2/ε3 group. These results may help generate future hypotheses that the off-line memory consolidation might be impaired in Alzheimer’s disease.

## Introduction

Müller and Pilzecker assumed that new memory takes time to be consolidated after encoding [1]. Many studies have shown that spontaneous brain activity after memory encoding is associated with memory consolidation. Single-unit recording animal study suggested that the spontaneous activity of the hippocampal place cells after learning is associated with memory consolidation [2]. With fairly good spatial and temporal resolution, RS-fMRI provides unique non-invasive technique to investigate the spontaneous activity of the human brain. Recent RS-fMRI studies have indicated an association of spontaneous brain activity with memory consolidation of recent scenarios [3]–[6]. These results support the system consolidation theory, which hypothesized that the MTL (mainly hippocampus and parahippocampus) is required for initial storage and recall and that the neo-cortex is considered as the area where remote memory is stored [7], [8]. Recent memory consolidation studies also suggest that the MTL may play multiple roles in memory consolidation [9]. However, the changed relationship or functional connectivity between remote brain areas by these RS-fMRI studies did not reveal whether the local spontaneous activity in a specific area (e.g., MTL or neo-cortex) was modulated by a preceding task. Only two studies have measured the assciation between local activity and memory consolidation in human. One study used perfusion MRI and found that the regional cerebral blood flow increased in hippocampal and temporal lobe regions after a learning task and that the increases correlated with the performance of surprised recall task [10]. Another very recent study from our group found that the local synchronization of spontaneous fMRI signal in the MTL increased after an episodic memory task in a group of participants having better performance in a later surprise retrieval task, but not in a group having worse performance [11].

The presence of APOE ε4 allele is related to increased risk of sporadic Alzheimer’s disease (AD) [12], [13]. Subtle episodic memory deficits are the earliest cognitive symptom of AD [14]. Structural MRI studies have reported significant atrophy of the MTL in AD patients [15], [16]. Functional MRI studies consistently showed abnormal MTL activation in patients with AD [17] and amnestic mild cognitive impairment (aMCI) [18]. Altered brain activations were also reported even in healthy carriers of APOE ε4 during memory encoding stage in the hippocampus [19], [20]. All these studies provided strong evidences that the MTL in AD or APOE ε4 carriers is associated with episodic memory deficit. Thus our first hypothesis was that the APOE ε4 healthy carriers probably have a different memory consolidation process in the MTL as compared with ε4 non-carriers.

Two previous RS-fMRI studies found memory consolidation-related changes in functional connectivity between brain areas associated with stimulus encoding. Albert et al. [5] showed that a resting state fronto-parietal network that is believed to be involved in visuomotor processing was modulated by motor learning, but not motor performance. Tambini et al. [6] showed that after a task with higher associative memory performance rather than poor memory performance, the magnitude of resting state functional connectivity between the hippocampal and lateral occipital complex was altered. The results from animal and human studies support the system consolidation theory, which hypothesized that the MTL is required for initial storage and recall and that the neo-cortex is considered as the area where remote memory is stored [7], [8]. Therefore, our second hypothesis was that the changes of local spontaneous activity related to off-line memory consolidation in the areas where picture encoding occurs might be different between APOE ε4 healthy carriers and non-carriers.

## Materials and Methods

### Participants

There were two sub-studies. The whole study was approved by the Institutional Review Board of the State Key Laboratory of Cognitive Neuroscience and Learning, Beijing Normal University. The first sub-study was an APOE genotype screening study. 1220 students at Beijing Normal University were enrolled by advertisement when they were taking a freshmen entrance physical examination. After they gave a written informed consent, 4 ml extra blood was obtained when they were undergoing blood sampling during the physical examination. They were told that DNA genotyping results would not be sent to them.

Genomic DNA was extracted from the peripheral blood leukocytes using salting out method [21]. 917 of the 1220 samples were genotyped for the APOE allele type using PCR-RFLP [22]. Six APOE genotypes were identified: ε2/ε2 (N = 1), ε2/ε3 (N = 52), ε2/ε4 (N = 13), ε3/ε3 (N = 799), ε3/ε4 (N = 51) and ε4/ε4 (N = 1).

It has been reported that APOE ε2 may exert protective effects [23]. It is therefore expected that effects of the ε2/ε3 vs. ε3/ε4 may be larger than those of ε3/ε3 vs. ε3/ε4. About one year later, 40 healthy Han Chinese students (aged 18 - 23 years of age), 20 for ε2/ε3 group and 20 for ε3/ε4 group, were chosen from the above sample while the sex was matched between the two groups. The number of participants were kept similar from each college of Beijing Normal University. All participants were right-handed and without a family history of AD by self report. None of the participants had current or past history of neurological or psychiatric disorders. All participants gave written informed consent for MRI scanning and they did not know their genotypes. 35 participants completed the Wechsler Adult Intelligence Scale-Revised for China (WAIS-RC). After the MRI scanning, they were all paid for their participation. One ε2/ε3 participant was excluded because of abnormal brain structure.

### Scanning Procedure

MRI data was acquired on a SIEMENS TRIO 3.0 Tesla scanner in Beijing Normal University. There were five scanning sessions:

1. The first RS-fMRI session (REST-1): 8 min (240 volumes). Participants were instructed to keep awake, close their eyes, and think of nothing in particular. Functional T2* blood oxygenation level-dependent (BOLD) images were obtained using an echo-planar imaging (EPI) sequence with 33 contiguous axial slices (thickness/gap = 3/0.6 mm, repetition time (TR) = 2000 ms, echo time (TE) = 30 ms, flip angle = 90°, matrix = 64×64, and a field of view (FOV) = 200×200 mm^2^).
2. Memory encoding task fMRI: 5 min (151 volumes). Participants were asked to press a button in their left or right hands by their index fingers to indicate that a picture was indoor or outdoor. Each picture appeared on center of the screen for 1500 ms. Participants were asked to make a decision in 2000 ms. The time between the end of a picture and the beginning of next picture was 500–6500 ms (average 3500 ms). In total 60 pictures (30 indoor and 30 outdoor) were presented in a pseudo-random order via E-prime software. To improve accuracy and the familiarity to the procedure, a short practice (10 different indoor/outdoor pictures) immediately before this task session was performed. Scanning parameters were the same as REST-1.
3. Structure MRI: approximately 10 min. We acquired T1-weighted sagittal three-dimensional images using magnetization-prepared rapid gradient echo (MPRAGE) sequence (128 slices, TR = 2530 ms, TE = 3.39 ms, slice thickness  = 1.33 mm, flip angle = 7°, inversion time = 1100 ms, FOV = 256×256 mm^2^ and in-plane resolution = 256×192).
4. The second RS-fMRI session (REST-2): 8 min (240 volumes). Instructions and scanning parameters of this session were the same as REST-1.
5. Memory retrieval task fMRI: two runs, each lasting 5 min (151 volumes). Immediately before this task, the participants were told there would be a test task. The instructions were presented on the screen and read by the experimenter. When a picture was presented, the participants had to press the button in their right hands if they had ever seen the picture during the encoding stage; otherwise, they had to press the button in their left hands. In total 120 pictures, 60 old and 60 new, and 30 indoor and 30 outdoor were presented. The reaction time (RT) was recorded. The scanning parameters and picture presentation times were the same as REST-1. In this session, only behavior data was analyzed.

Participants were not told that there would be a retrieval task until immediately before this retrieval task. Accordingly, the possibility of intentional memory performance was minimized during the two RS-fMRI sessions.

### Pictures for Task Presentation

Encoding and retrieval sessions included totally 120 color pictures, half depicting indoor scenes and the other half depicting outdoor scenes. In order to make the error rate varies fairly large across participants (i.e., not too easy or hard to remember), we selected 120 pictures from a set of 180 pictures using a pilot behavioral experiment. Of these 180 pictures, 69 were neutral pictures from the International Affective Picture System (IAPS) [24] and the rest were obtained from the internet.

All these pictures could be easily classified into indoor or outdoor. All pictures were formatted in 640×480 pixels and were presented via E-prime software. We recruited an additional 12 college students to perform the pilot behavioral experiment. This pilot behavioral experiment had an encoding stage and a retrieval stage with a resting interval of approximately 20 minutes. The instructions and task presentation of the pilot experiment were the same as the fMRI task. The participants were not told it was a memory task before the retrieval stage. After the indoor or outdoor judgment task, they were just told to take a break to wait for another behavioral experiment. These participants were divided into 3 groups, each group (4 participants) encoding 60 images (30 indoor and 30 outdoor). The retrieval stage was the same as that of fMRI task, i.e., 60 old pictures and 60 new pictures. For each of the 180 old pictures, there were 4 participants. Therefore the hit number would be 0, 1, 2, 3, and 4 for each picture. For pictures with a hit score of 0 or 4 (i.e., no or all participants could remember correctly), only a small part of these pictures was chosen as the fMRI task pictures. But for pictures with a hit score of 1, 2, or 3, large part of these pictures was chosen. Finally, 120 pictures were selected for the fMRI study. The above procedure for picture collection was the same one as in our previous paper published very recently [11].

### Behavior Data Analysis

D-prime is a statistic used in signal detection theory and calculated as: d-prime = Z (hit rate) - Z (false alarm rate) [25]. Z is the standard Z score (minus mean and then divided by the standard deviation). A higher d-prime means a larger difference between the hit rate and correct rejection, i.e., a better recognition memory performance here.

### fMRI Data Preprocessing

The first 10 time points were discarded to remove the initial transient effects and to allow participant get used to the scanner noise. Then RS-fMRI data were processed using SPM8 (http://www.fil.ion.ucl.ac.uk/spm) and DPARSF [26] (http://www.restfmri.net), including slice timing, realignment and normalization. All functional images were normalized into Montreal Neurology Institute (MNI) space in three stages. First, the functional images of each participant were co-registered with their own high-resolution structural T1 image. Second, the spatial normalization parameters of T1 image were estimated by segmentation. Lastly, the parameters were applied to the functional images. Then REsting-State fMRI data analysis Toolkit [27] was used for removing the linear trend and temporal filtering (0.01–0.08 Hz) of the time courses.

### Region of Interest (ROI) Definition

**Figure 1:**
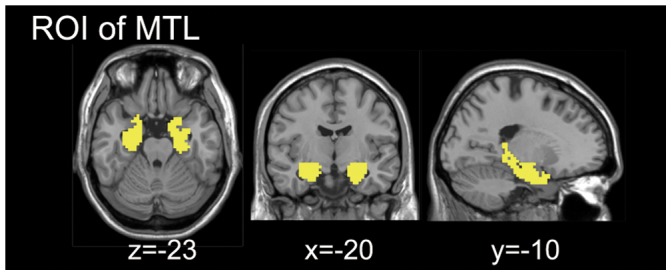
The medial temporal lobe (MTL) ROI. The MTL ROI from AAL, including the bilateral hippocampus and parahippocampal regions.

ROIs were defined by AAL template [28]. The medial temporal lobe (MTL) ROI includes bilateral hippocampus and parahippocampal region (Figure 1). The encoding-related ROIs were defined by the activation map of encoding pictures (*p*<0.05, cluster size >270 mm^3^).

### RS-fMRI Data Analysis

We used ReHo [29] to measure the local synchronization of spontaneous brain activities, which was estimated for each participant by computing Kendall’s coefficient of concordance (KCC) of the time series of 27 nearest neighboring voxels. Compared with functional connectivity, ReHo requires no a priori definition of ROIs, and can provide local synchronization of spontaneous activity. ReHo has been used in AD and MCI researches [30], [31], and ReHo has been found to be correlated with some behavior performance, such as stop signal reaction time [32]. REST software [27] was used to build individual ReHo maps. To quantify changes of the memory consolidation-related spontaneous brain activity, we used the ReHo ratio below:
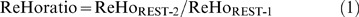
(1)


For each participant, ReHo of REST-2 was divided by ReHo of REST-1 in a voxel-wise manner to get the map of ReHo ratio. And then ReHo ratio maps were smoothed using a 4 mm FWHM Gaussian kernel. ReHo ratio between the two APOE groups were compared using the two-sample T-tests voxel by voxel in each ROI. AlphaSim, a program based on Monte Carlo simulation and implemented in AFNI (http://afni.nimh.nih.gov) was used for multiple comparison correction within each ROI (i.e., small volume correction, SVC). AlphaSim has been implemented in REST software [27]. The corrected *p*<0.05 corresponds to a combination of uncorrected *p*<0.05 and cluster size >567 mm^3^ in the MTL ROI and a combination of *p*<0.05 and cluster size >1566 mm^3^ in the encoding ROI. Finally, correlation analysis between ReHo ratio and recognition memory performance was performed in each group.

To remove potential effects of encoding activation on the memory consolidation-related activity in the MTL, we performed ANCOVA to regress out the task-evoked activation (mean beta estimate of each voxel).

### Encoding fMRI Data Analysis

Encoding fMRI data pre-processing was similar to resting fMRI data, including slice timing, realignment, normalization and smoothing (4 mm FWHM). Individual statistical models were constructed in SPM8 for each participant using a general linear model (GLM). Statistical contrasts were constructed to compare picture encoding with zero. In the second level analysis, one-sample T-tests were applied to identify regions activated by GLM. The positive activation map (*p*<0.05, cluster size >270 mm^3^) obtained by the one-sample T-tests was taken as encoding-related ROIs for resting-state analysis. We also compared the activation between the two groups using two-sample T-test. Brain regions above combination threshold of *p*<0.05 and cluster size >2295 mm^3^ were considered as significantly different between groups.

## Results

### Behavioral Results

The demographic and behavioral data are listed in Table 1. There were no significant differences in age, gender, IQ score and d-prime score (all *p*’s >0.05).

**Table 1:** Demographic data and behavior performance of two APOE groups.

	Sex (male:female)	Age	IQ full scale	d-prime
APOE ε2/ε3	9∶10	20.5±0.8	126.79±7.58	2.47±0.70
APOE ε3/ε4	10∶10	20.8±0.9	126.53±7.79^a^	2.54±0.62

a Only 15 participants tested IQ in APOE ε3/ε4 group.

### RS-fMRI Results in MTL

**Figure 2:**
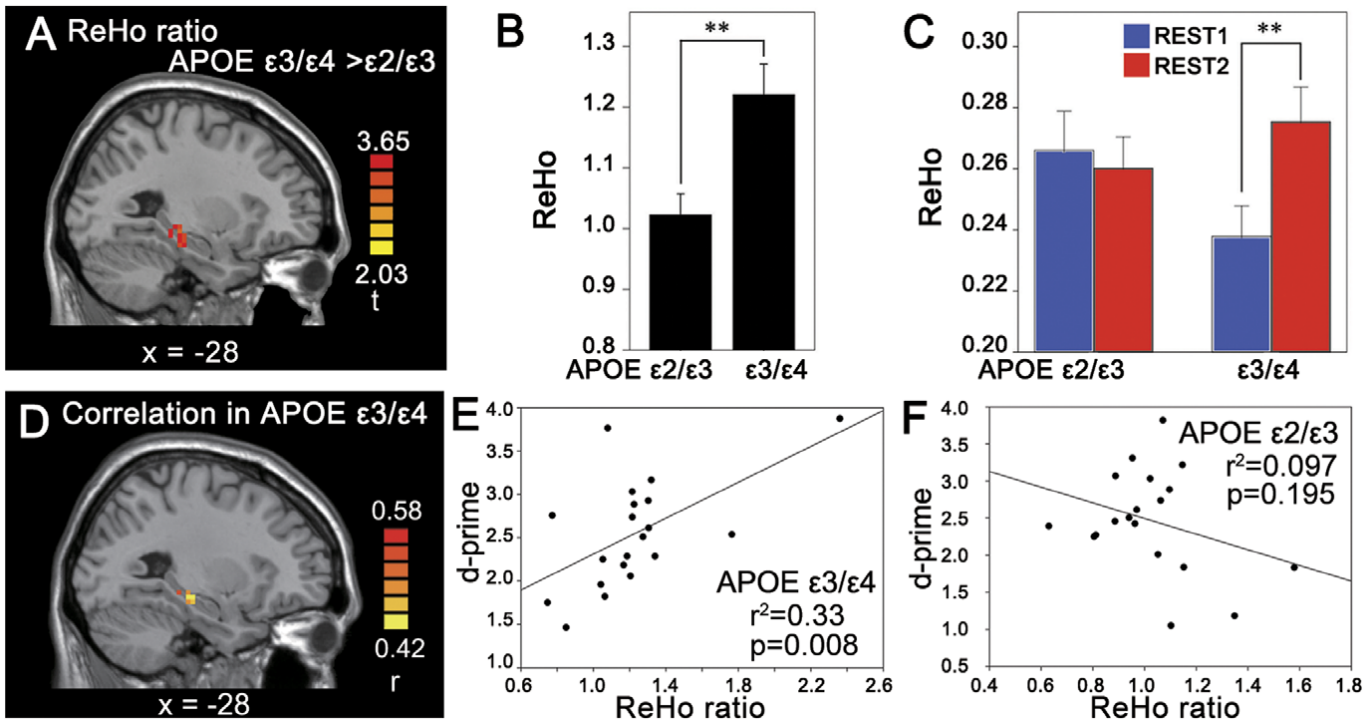
Memory consolidation-related changes in the MTL. (A): Significant higher ReHo ratio in APOE ε3/ε4 group than ε2/ε3 group. (B): The ReHo ratio (mean and standard error) averaged from the cluster shown in (A). (C): The ReHo value (mean and standard error) averaged from the cluster in the MTL of REST1 and REST2, respectively, for each group. (D): Correlation map between ReHo ratio and recognition memory performance (d-prime) in the MTL cluster as shown in Figure 2A in APOE ε3/ε4 group (n = 20). (E): Regression plot demonstrating the correlation between d-prime and ReHo ratio extracted from a voxel in the MTL (−27, −27, −12) in two groups separately. **: *p*<0.01.

In MTL ROI, two sample t-test showed that the ReHo ratio in APOE ε3/ε4 group was significantly larger than that in APOE ε2/ε3 group (*p*<0.05, two-tailed, small volume correction (SVC)) (Figure 2A). Paired t-tests revealed that the APOE ε3/ε4 group showed significant higher ReHo in REST-2 than in REST-1 in MTL ROI (*t* = 3.789, *p* = 0.001), but no significant ReHo difference was observed between REST-1 and REST-2 in the APOE ε2/ε3 group (*t* = 0.569, *p* = 0.576) (Figure 2B and 2C, respectively). Furthermore, the ReHo ratio showed significantly positive correlation with d-prime, an index of recognition memory performance, obtained from the retrieval stage in APOE ε3/ε4 group (*p*<0.05, SVC), but the correlation was not significant in APOE ε2/ε3 group in this ROI (Figure 2D). It should be noted that this correlation analysis was performed within the cluster in which the voxels showed significant difference in ReHo ratio between the two groups. Using ANCOVA to regress out the task-evoked activation, the significant difference in ReHo ratio between the two groups remained (*p*<0.05, SVC).

### RS-fMRI Results in Neo-cortex

**Figure 3:**
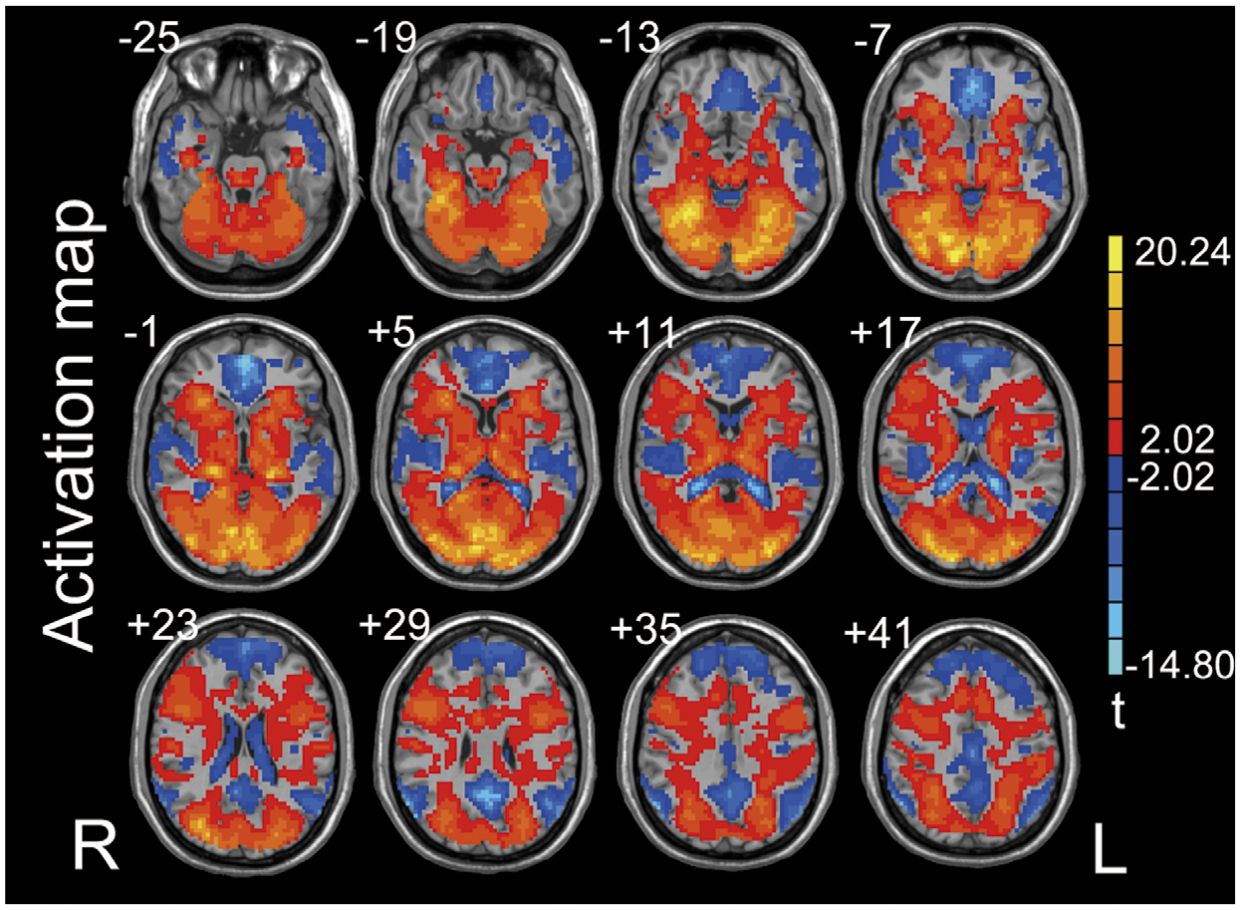
Activation map of encoding pictures (*p*<0.05, cluster size >270 mm^3^) of all participants. The positive activation areas were defined as encoding-related ROIs for ReHo ratio comparison between the two groups.

The activated areas during the encoding task were identified as the encoding-related ROIs (p<0.05, cluster size >270 mm^3^, Figure 3).

**Figure 4:**
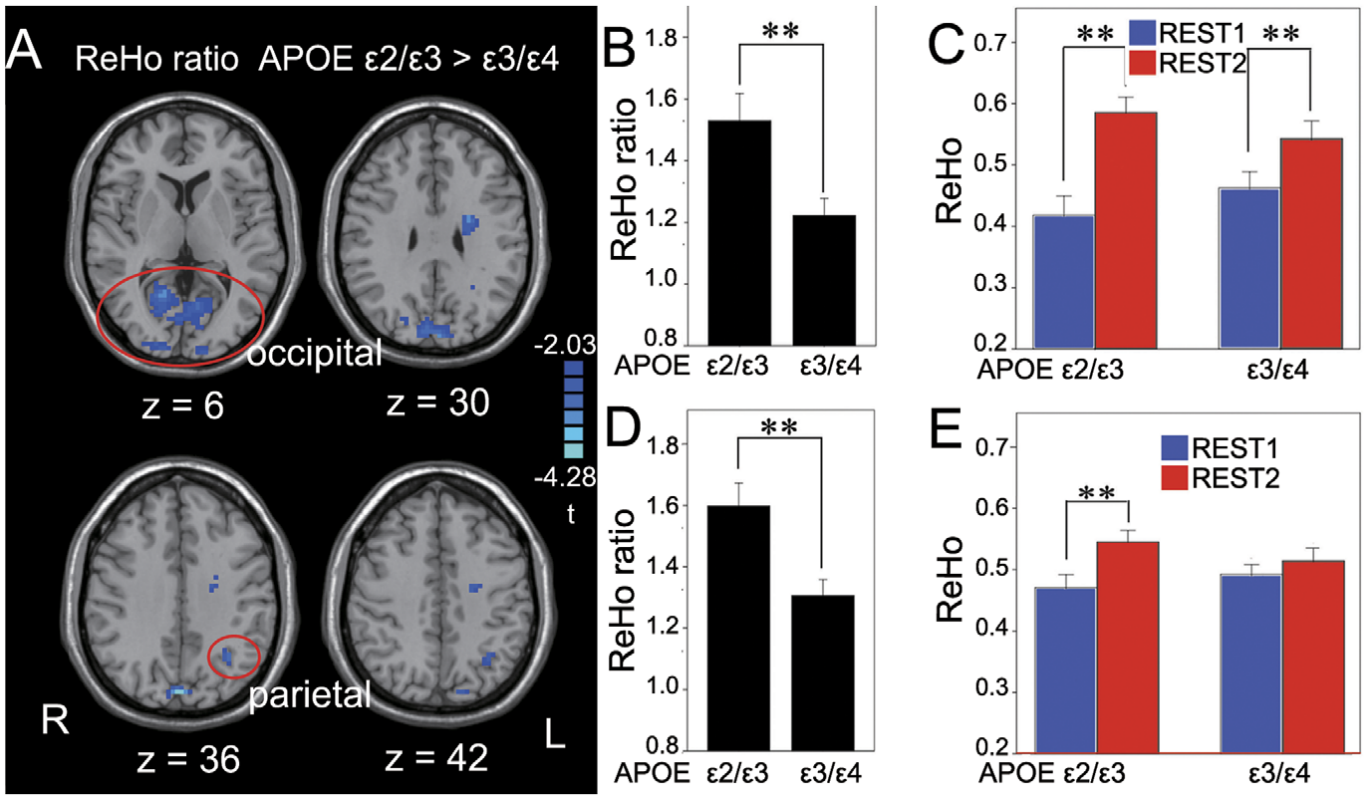
Memory consolidation-related changes in the encoding-related brain areas. (A): Significant higher ReHo ratio in APOE ε2/ε3 group than ε3/ε4 group in the occipital cortex bilaterally (upper) and left superior parietal lobule (lower). (B) and, (D): The ReHo ratio (mean and standard error) averaged from the clusters shown in the upper part and low part, respectively, of (A). (C) and (E): The ReHo value (mean and standard error) averaged from the clusters in the upper part and lower part, respectively, of (A) for each group. **: *p*<0.01.

**Figure 5:**
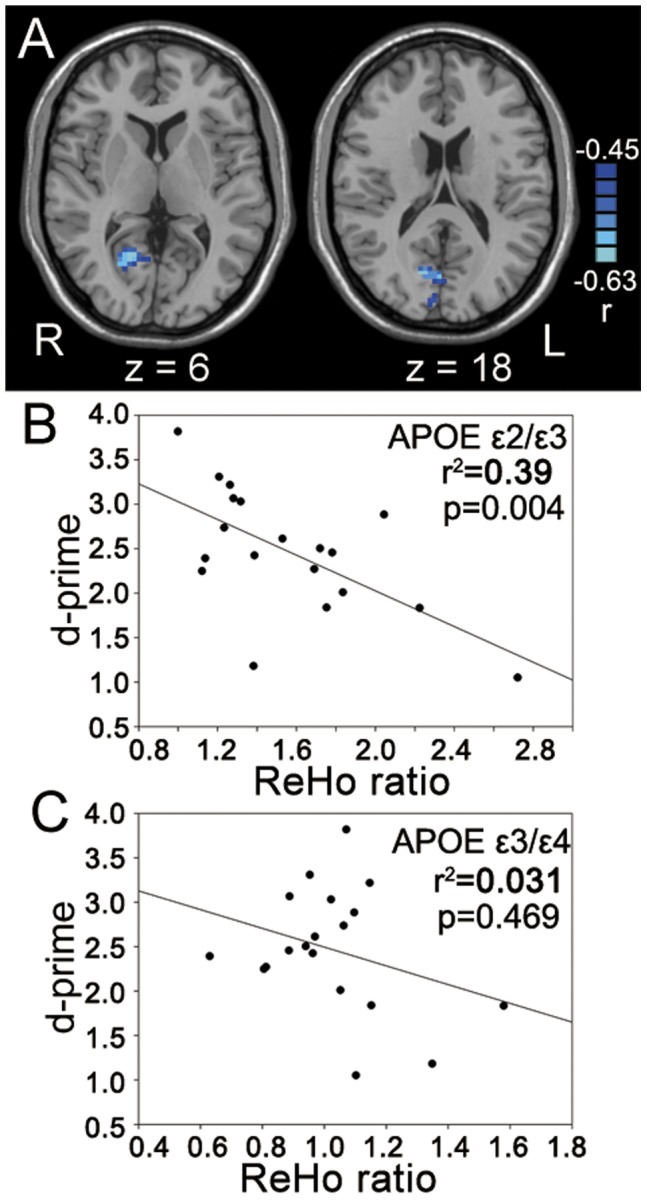
Correlation map between ReHo ratio and d-prime in the visual cortex (upper part of **Figure 4A**). (A): Significant correlation (*p*<0.05, SVC) between ReHo ratio and recognition memory performance (d-prime) in APOE ε2/ε3 group (n = 19). (B) and (C): Regression plot demonstrating the correlation between d-prime and ReHo ratio extracted from a voxel in the occipital ROI (15, −60, 9) in two groups separately.

In the occipital and parietal areas, APOE ε3/ε4 group showed significantly lower ReHo ratio than APOE ε2/ε3 group (*p*<0.05, SVC) (Figure 4A & Table 2). Paired t-tests showed higher ReHo of REST-2 than that of REST-1 in the two groups in the occipital area (APOE ε2/ε3: *t* = 6.928, *p* = 0.001; APOE ε3/ε4: *t* = 3.401, *p* = 0.003) (Figure 4C) but in the parietal area, only in APOE ε2/ε3 group (*t* = 5.134, *p* = 0.001) (Figure 4E). Furthermore, in the occipital area, ReHo ratio showed a significantly negative correlation with the recognition memory performance (d-prime value) in APOE ε2/ε3 group (*p*<0.05, SVC), but showed no significant correlation in APOE ε3/ε4 group (Figure 5). It should be noted that this correlation analysis was performed within the visual cortex in which the voxels showed significant difference in ReHo ratio between the two groups (upper part of Figure 4A). The above results may indicate that the encoding area is more involved in memory consolidation in APOE ε2/ε3 group during a relatively early stage of memory consolidation than done in APOE ε3/ε4 group.

**Table 2:** Clusters with significantly different ReHo Ratio between the two APOE groups in Encoding-related ROI.

Direction of response	Hemisphere	Subregion	Brodmann’s area	Cluster size	Coordinates of peak voxel	*t* value of peak voxel
APOE ε3/ε4< APOE ε2/ε3	R	Cuneus, lingual gyrus	BA 18, 19, 30	37071 mm^3^	3, −84, 36	−4.282
	L	White matter	NA	1917 mm^3^	−24, −6, 33	−3.295
	L	Superior parietal lobule	BA 7	1782 mm^3^	−30, −57, 33	−3.080

## Discussion

In the current study, we compared the ReHo change after episodic memory encoding, and found the difference between APOE ε3/ε4 group and APOE ε2/ε3 group in MTL and encoding-related brain areas. The results of our study showed that APOE ε4 carriers may have a different episodic memory consolidation process, and this memory consolidation process is not affected by encoding effects. Our study of memory consolidation showed the gene effects on memory consolidation and provided a new sight in memory consolidation process in memory-related disease. This study also suggests that the RS-fMRI may serve as an effective approach for investigating spontaneous memory consolidation of the human brain.

ReHo is a measurement of local synchronization of BOLD signals. ReHo had been used in many brain disorder studies. In epilepsy studies, ReHo had detected the abnormally increase local synchronization within the epileptic focus, such as increased ReHo in MTL of temporal lobe epilepsy patients [33] and increased ReHo in the thalamus of generalized tonic-clonic seizures [34]. Based on these evidences, the increased ReHo after a memory task may suggest an increased local synchronization of neural activity related to off-line memory consolidation.

Although APOE ε4 young healthy carriers showed significantly greater ReHo ratio than non-carriers in MTL, the two groups did not show significant differences in recognition memory performance (i.e., d-prime, *p* = 0.73), being consistent with a previous study which did not find significant differences in memory performance between young (<50 years old) APOE ε4 healthy carriers and non-carriers [13]. The increased ReHo ratio of spontaneous activity in APOE ε4 healthy carriers may indicate a compensative off-line memory consolidation process, which may have resulted in similar recognition memory performance for the two groups during subsequent memory retrieval stage. The significantly positive correlation between ReHo ratio and recognition memory performance observed only in ε4 carriers also supports this interpretation. The decreased memory consolidation-related spontaneous activity may lead to worse memory performance in aged APOE ε4 carriers.

There is a possibility that the different consolidation effect after task was a direct consequence of different encoding activation of the preceding task between the two groups. Previous fMRI studies comparing the activation during encoding between successful encoding and failure encoding showed that the activation of encoding can predict the memory performance (subsequent memory effect) [19], [35]. Greater functional activation in MTL during encoding has been reported in young APOE ε4 healthy carriers than non-carriers [19], and our results also showed greater encoding related activation in the right MTL in the APOE ε3/ε4 group than in APOE ε2/ε3 group. However, the significant difference in ReHo ratio between the two groups remained after the task-evoked activation was regressed out. This result suggests that the consolidation effect is largely independent of the encoding activation. The different activation during encoding does not account for the different memory consolidation in the MTL. Therefore, it is essential to focus on memory consolidation process.

The results in neo-cortex also showed that the local spontaneous activity in the areas where picture encoding occurs was modulated by the task. Albert et al. showed that a resting state fronto-parietal network that is believed to be involved in visuomotor processing was modulated by motor learning, but not by motor performance [5]. Tambini et al. showed that after a task with higher associative memory performance rather than poor memory performance, the magnitude of resting state functional connectivity between the hippocampal and lateral occipital complex was altered [6]. The results from animal and human studies support the system consolidation theory, which hypothesized that the MTL (mainly hippocampus and parahippocampus) is required for initial storage and recall and that the neo-cortex is considered as the area where remote memory is stored [7]–[9]. The role of the visual cortex in memory consolidation remains unclear. In addition to visual perception and representation, studies have shown that the visual cortex is important for visual recognition memory and visual learning [36]. An RS-fMRI study showed that the changes in functional connectivity between the hippocampus and the visual cortex were involved in memory consolidation [6]. Our results provide evidence of association between the local spontaneous activity in the visual cortex and episodic memory consolidation. The difference in ReHo ratio between the two genotype groups in the visual cortex and hippocampus may suggest a different mechanism of memory consolidation for APOE ε4 carriers.

Several limitations should be addressed. First, the off-line memory consolidation is a time dependent and dynamic process [37]. We only collected the data of 8–16 min after encoding. The dynamic course of off-line memory consolidation for a specific brain area needs to be further investigated. System consolidation suggests the transfer between the MTL and neo-cortex during consolidation. In our study, we just focused on the activity in the MTL and neo-cortex separately. Second, we did not collect neuropsychological data of episodic memory tests which are widely used in MCI and AD studies while we only performed correlation analysis between ReHo ratio and retrieval performance. Third, we did not include APOE ε3/ε3, the most common genotype in population, and therefore, the differences in ReHo between APOE ε2/ε3 and ε3/ε4 might not be attributed to the differences in ReHo between ε4 carriers and other genotype carriers. Finally, abnormal activation during memory encoding stage has been widely reported in aMCI and AD patients [17], [38], [39]. However, no imaging study has been carried out to investigate the spontaneous memory consolidation stage in aMCI or AD patients. The current results may shed new light on the mechanism of episodic memory deficit in AD. It would be interesting to determine if there is a vulnerable period of off-line memory consolidation for aMCI or early AD patients. If so, a cognitive therapy might be developed.

### Conclusion

In summary, we provide the first demonstration of memory consolidation process of different APOE carriers in the specific areas (such as MTL and encoding related areas) by using RS-fMRI. We found that both in MTL and encoding related neo-cortex showed memory consolidation related activity change after episodic memory encoding. And the mechanism of off-line episodic memory consolidation is different in APOE ε4 carriers.
